# Prognostic significance of infarct core pathology in ST-elevation myocardial infarction survivors revealed by quantitative T2-weighted cardiac magnetic resonance

**DOI:** 10.1186/1532-429X-17-S1-O54

**Published:** 2015-02-03

**Authors:** David Carrick, Caroline Haig, Samuli M Rauhalammi, Nadeem Ahmed, Ify Mordi, Margaret McEntegart, Mark Petrie, Hany Eteiba, Stuart Hood, Stuart Watkins, Mitchell Lindsay, Ahmed Marous, Aleksandra Radjenovic, Ian Ford, Niko Tzemos, Keith G Oldroyd, Colin Berry

**Affiliations:** 1 Golden Jubilee National Hospital, Clydebank, UK; 2 Institute of Cardiovascular and Medical Sciences, University of Glasgow, Glasgow, UK; 3 Robertson Center for Biostatistics, University of Glasgow, Glasgow, UK

## Background

Myocardial transverse relaxation time (T2, ms) is a fundamental magnetic property of tissue that is related to water content and mobility. The pathophysiological and prognostic importance of native myocardial T2 in acute ST-elevation myocardial infarction (STEMI) patients is unknown. We aimed to assess the clinical significance of native T2 within the infarct core using cardiac magnetic resonance (CMR) imaging.

## Methods

We performed a prospective single center cohort study in reperfused STEMI patients who underwent CMR 2 days and 6 months post-MI. T2-weighted CMR (investigational prototype T2-prepared TrueFisp sequence) was measured in myocardial regions-of-interest. The infarct territory and microvascular obstruction were depicted with late gadolinium enhancement CMR. All-cause death or heart failure hospitalization was a pre-specified outcome that was assessed during follow-up.

## Results

324 STEMI patients (mean±SD age 59±12 years, 237 males, 121 with anterior STEMI) gave informed consent and had CMR (14 July 2011 - 22 November 2012). All 324 had follow-up assessments (median duration 860 days). Infarct size was 18 ±14% of LV mass. One hundred and sixty four (51%) patients had late microvascular obstruction whereas 197 (61%) patients had an infarct core revealed by native T2. Native T2 within the infarct core (53.9±4.8) was higher than in the remote zone (49.7±2.1 ms; p<0.01) but lower than in the area-at-risk (62.9±5.1 ms) (p<0.01). In multivariable linear regression, native T2 in the infarct core was negatively associated with heart rate, Killip class, and peak neutrophil count at presentation (all p<0.05).

Baseline T2 core (ms) was univariably associated with LVEF (0.31 (0.04, 0.58); p=0.023). Baseline T2 core was not associated with LVEF or volumes at 6 months.

Thirty (10.4%) patients died or experienced a heart failure event. These events included 5 cardiovascular deaths, 3 non-cardiovascular deaths and 22 episodes of heart failure (Killip Class 3 or 4 heart failure (n=20) or defibrillator implantation n=2). T2-core (ms) was associated with all-cause death or heart failure hospitalization (hazard ratio 0.786, 95% CI 0.658, 0.939; p=0.008) including after adjustment for LVEF at baseline (p=0.017) or LV end-diastolic volume at baseline (p=0.009).

## Conclusions

Infarct core revealed by native T2 was common and independently associated with all-cause death or heart failure hospitalization post-discharge.

## Funding

N/A.

**Figure 1:**
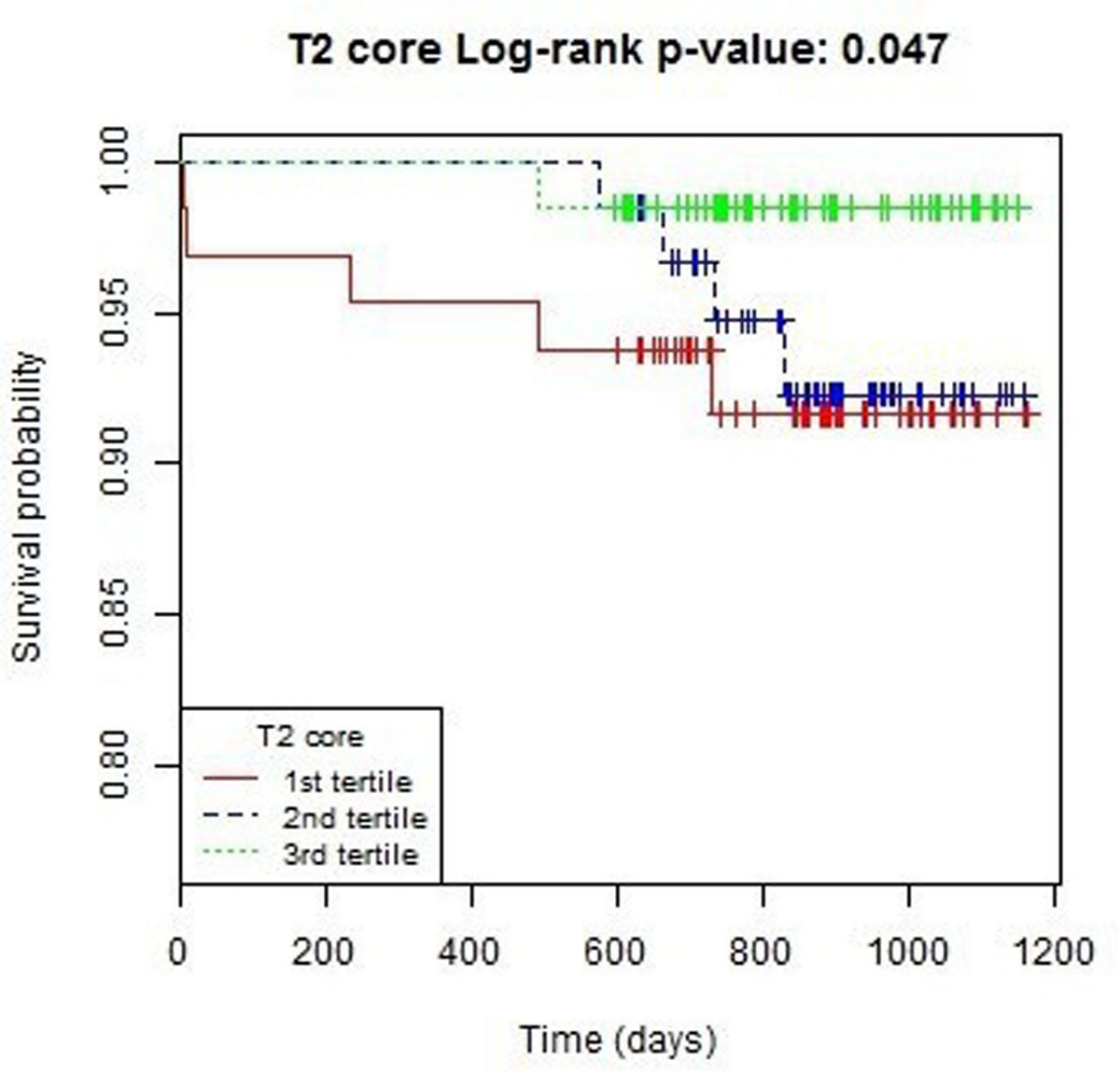
Kaplan-Meier survival curves grouped according to baseline infarct core T2 (lowest T2 tertile vs.tertiles 2 and 3) and all-cause death or heart failure (n=39) from and including the index admission to the end of follow-up (censor time 839 (598 to 1099) days).

